# Gait speed correlates in a multiracial population of community-dwelling older adults living in Brazil: a cross-sectional population-based study

**DOI:** 10.1186/1471-2458-13-182

**Published:** 2013-02-28

**Authors:** Cintia Regina Ruggero, Tereza Lofredo Bilton, Luiza Faria Teixeira, Juliane de Lemos Armada Ramos, Sandra Regina Alouche, Rosangela Correa Dias, Monica Rodrigues Perracini

**Affiliations:** 1 Master’s and Doctoral Programs in Physical Therapy, Universidade Cidade de São Paulo, Sao Paulo, Brazil; 2 Human Science and Health College, Pontificia Universidade Catolica, Sao Paulo, Brazil; 3 Faculty of Medicine, State University of Campinas, Sao Paulo, Brazil; 4 Physiotherapy Department, Federal University of Minas Gerais, Belo Horizonte, Brazil; 5 Visiting Research Fellow at Sydney University and The George Institute for Global Health, Sydney, Australia; 6 Rua Cesareo Galeno, 448, Sao Paulo, Tatuape, Brazil

**Keywords:** Gait speed, Aged, Aged health, Urinary incontinence, Physical performance, Cross-sectional studies

## Abstract

**Background:**

Gait speed is a strong predictor of a wide range of adverse health outcomes in older adults. Mean values for gait speed in community-dwelling older adults vary substantially depending on population characteristics, suggesting that social, biological, or health factors might explain why certain groups tend to self-select their gait speed in different patterns. The vast majority of studies reported in the literature present data from North American and European populations. There are few population-based studies from other regions with a different ethnicity and/or social and health conditions. To address this, the present study identified the mean usual and fast gait speeds in a representative multiracial population of community-dwelling older adults living in a developing country, and explored their association with sociodemographic, mental and physical health characteristics.

**Methods:**

This was a cross-sectional population-based study of a sample of 137 men and 248 women, aged 65 years and over. Usual gait speed and fast gait speed were measured on a 4.6 m path. Participants were classified into slow, intermediate, and faster groups by cluster analysis. Logistic regression analysis was used to estimate the independent effect of each factor on the odds of presenting with a slower usual and slower fast gait speeds.

**Results:**

Participants had a mean (SD) usual gait speed of 1.11 (0.27) m/s and a mean fast gait speed of 1.39 (0.34) m/s. We did not observe an independent association between gait speed and race/ethnicity, educational level, or income. The main contributors to present a slower usual gait speed were low physical activity level, stroke, diabetes, urinary incontinence, high concern about falling, and old age. A slower fast gait speed was associated with old age, low physical activity, urinary incontinence and high concern about falling.

**Conclusion:**

A multiracial population of older adults living in a developing country showed a similar mean gait speed to that observed in previously studied populations. The results suggest that low physical activity, urinary incontinence and high concern about falling should not be neglected and may help identify those who might benefit from early intervention.

## Background

Gait speed is a strong predictor of a wide range of outcomes in older adults [1], including mortality [2], falls and fractures [3,4], hospitalization [5], need of a caregiver [6], functional disability of the lower limbs [7], limited activities of daily living [5], and cognitive decline [8,9].

The decrease in usual gait speed associated with increasing age [10,11] is thought to be around 0.013 m/s/year [12], and 0.027 m/s/year for fast gait speed [13] and is considered to mark a decline in functional reserve, which might be explained by cumulative age-related body changes, disease burden, or the presence of subclinical conditions such as atherosclerosis or chronic inflammation [14-16].

A cut-off of 1.0 m/s for usual gait speed identifies slower older adults with a high risk of negative health outcomes, such as persistent lower extremity limitation, hospitalization, and death [1,7]. Mean values for usual gait speed in community-dwelling older adults vary substantially; for example, from 0.56 m/s in a Hispanic American population to 1.19 m/s in a population of men (mainly White Americans) [2]. These differences in walking speed may reflect not only a multi-systemic impairment in health status, traduced by biological dysfunctions (for example, cognitive, musculoskeletal, neural) such as dynapenia [17], diminished cutaneous sensitivity and decreased nerve conduction velocity [18] and brain neuronal loss and presence of white matter lesions [19,20], but may be also influenced by racial differences [21], psychological and socioeconomic conditions such as a high concern about falling [22], low educational level [23] and low employment grade [24]. Therefore, social, biological or health factors might explain why certain groups tend to self-select their gait speed (e.g., usual or fast) in different patterns, but the significance of each factor within a multifactorial approach has not been fully explored. In addition, the vast majority of studies reported in the literature present data from North American and European populations [1,2]. Few population-based studies have been conducted in other regions with different ethnicity and social and health conditions. To address this, the aim of this study was to investigate the mean usual and fast gait speeds in a representative multiracial population of community-dwelling older adults living in Brazil, and to explore its association with sociodemographic, mental and physical health, and physical functioning. Identifying the factors associated with slower gait speed will help us to target vulnerable older adults who may benefit from early intervention.

## Methods

### Study design

Data were obtained using a cross-sectional population-based study based on the FIBRA Network Study (Frailty among Brazilian Older Adults).

### Setting

Elderly participants living at home in an urban area were enrolled through a process of random cluster sampling of census regions. Data were collected from March, 2009 to April, 2010.

### Participants

Both male and female participants aged 65 or older were included. Exclusion criteria were based upon the methodological recommendations proposed by Ferrucci et al. [25]: 1) severe cognitive impairment according to the Mini-Mental State Examination (MMSE), adjusted for education level [26]; 2) inability to walk (either temporarily or permanently); 3) localized loss of strength and aphasia due to severe stroke; 4) Parkinson’s disease (either severe or unstable); 5) severe hearing or visual impairment; and 6) terminal illness.

Each participant was instructed about the objectives and research procedures and all provided signed informed consent. This study was approved by the Ethics Committee at the Pontificia Universidade Catolica de Sao Paulo (protocol number 269/2007).

### Measures and instruments

Participants were evaluated by trained research assistants in two phases: the first phase consisted of a face-to face interview using a multidimensional structured questionnaire and lasting between 40 and 120 minutes. The second phase comprised a battery of physical function tests.

The study outcome variables were usual and fast gait speed (m/s), obtained by dividing the distance travelled (4.6 m) by the time taken to cover that distance using a stopwatch (Cronobio® model SW2018). The mean value of three trials was used for data analysis. The study incorporated a distance of 2 m for acceleration and a further 2 m for deceleration. The participants were wearing their usual footwear and used a walking aid/device as needed. Gait speed was assessed at a local community facility service in a well-lit room and on a flat surface.

### Sociodemographics

The variables selected were gender, age group (65–69, 70–74, and 75+) and educational level (illiterate or 1 or more years of education/study). Monthly house-hold income was measured in multiples of the minimum wage (US$ 290 in 2009) as follows: 0.0–1.0, 1.1–3.0, and ≥ 3.1. Race and ethnicity were classified as white, black, pardo (or brown) and “other” (indigenous and yellow).

### Mental health status

The MMSE score was used as a global test of cognitive function [26]. Symptoms of depression were assessed using the short version of the Geriatric Depression Scale (GDS-15) [27]; a score of ≥ 5 points was considered positive. The Falls Efficacy Scale International (FES-I) was used to assess the concern about falling or fear of falling when performing daily activities [28]. The total score can range from 16 (no concern/fear) to 64 (extreme concern/fear); a cut-off point of ≤ 22 was used in this study [29].

### Physical health

Self-rated health was categorized as very good/good, fair, or poor/very poor. The presence and number of chronic diseases or health conditions diagnosed by a doctor in the last 12 months was also documented (e.g., heart disease, high blood pressure, stroke, diabetes, arthritis, depression, osteoporosis and urinary incontinence). Patients were categorized as having no disease, one or two diseases, or more than three diseases. Polypharmacy was defined as the use of four or more regular medications during the last 3 months. Self-reported falls and recurrent falls (two or more) in the previous year were also documented and categorized dichotomously. Participants self-reported their fatigue levels by answering two questions taken from the Center for Epidemiologic Studies-Depression Scale (CES-D) [30], which were related to their levels of perceived exertion and abandonment of activities over the previous week. Fatigue was considered to be a factor when the participant responded positively to a frequency of “most often” (3 or 4 days) or “always” (5 to 7 days).

### Physical function

The Katz Index [31] was used to measure participants’ self-reported independence in activities of daily living (bathing, dressing, toileting, transfers, continence, and feeding). Participants scored each activity as follows: 0 = no supervision, direction, or personal assistance required; 1 = supervision, direction, personal assistance or total care required [22]. The scores were then added together to provide a measure of independence. The median score was used for categorization (independent or dependent) in the present study.

Physical activity level was measured using a short version of the Minnesota Leisure Time Activities Questionnaire (Q-MLTPA) [32]. Participants were questioned with regard to activities carried out, and the mean duration (in minutes) of each activity, over the previous 2 weeks. Energy expenditure was measured in kilocalories/minute (0.0175 Kcal × min-1 × MET × body weight in kg). The lowest quintile of total energy expenditure (low physical activity level) for each individual (in Kcal/week) was used to classify the participants [32].

### Anthropometrics

Standing height and weight were measured using a flexible steel rule fixed to the wall and a digital portable weighing device, respectively and were used as continuous variables.

### Statistical analysis

The K-means clusters method was used to establish the cut-off points for usual and fast gait speed (m/s) [33]. For usual gait speed, participants were categorized as slower (<0.91m/s); intermediate (0.91 to 1.26 m/s) and faster (>1.26 m/s). For fast gait speed, participants were categorized as slower (<1.09 m/s), intermediate (1.09 to 1.57m/s) and faster (>1.57m/s).

The groups were compared using analysis of variance (ANOVA) and the Chi-squared test to present the characteristics of the population studied in terms of distribution within the slower, intermediate and faster groups (for both usual gait speed and fast gait speed). A stepwise multivariate logistic regression analysis was performed to estimate the independent effects of each demographic, mental and physical health, and physical function on the odds of presenting with a slower usual or slower fast gait speed, when compared to the elderly who were in the intermediate or faster groups. A screening criterion of p< 0.05 was used to select independent variables for entry into the multiple analyses. Confounders were identified by a change of > 20% in the parameter estimate (β coefficient). The influence of stature on both usual and fast gait speed was also examined, because population was multiracial with varied anthropometric profiles, which may influence gait speed. Odds ratios with lower and upper 95% confidence intervals (95% CI) and p-values were reported. The fit of the multiple logistic regression models was evaluated using the Hosmer-Lemeshow goodness-of-fit test. Discrimination (the ability to distinguish those who presented with a slower gait speed from those who did not) was quantified using the area under the receiver operating characteristic curve (ROC curve) [1] within a 95% CI. All tests were two-tailed and p < 0.05 was considered statistically significant. The statistical package used for all analysis was SPSS® 17 (SPSS Inc., Chicago, IL).

## Results

**Figure 1:**
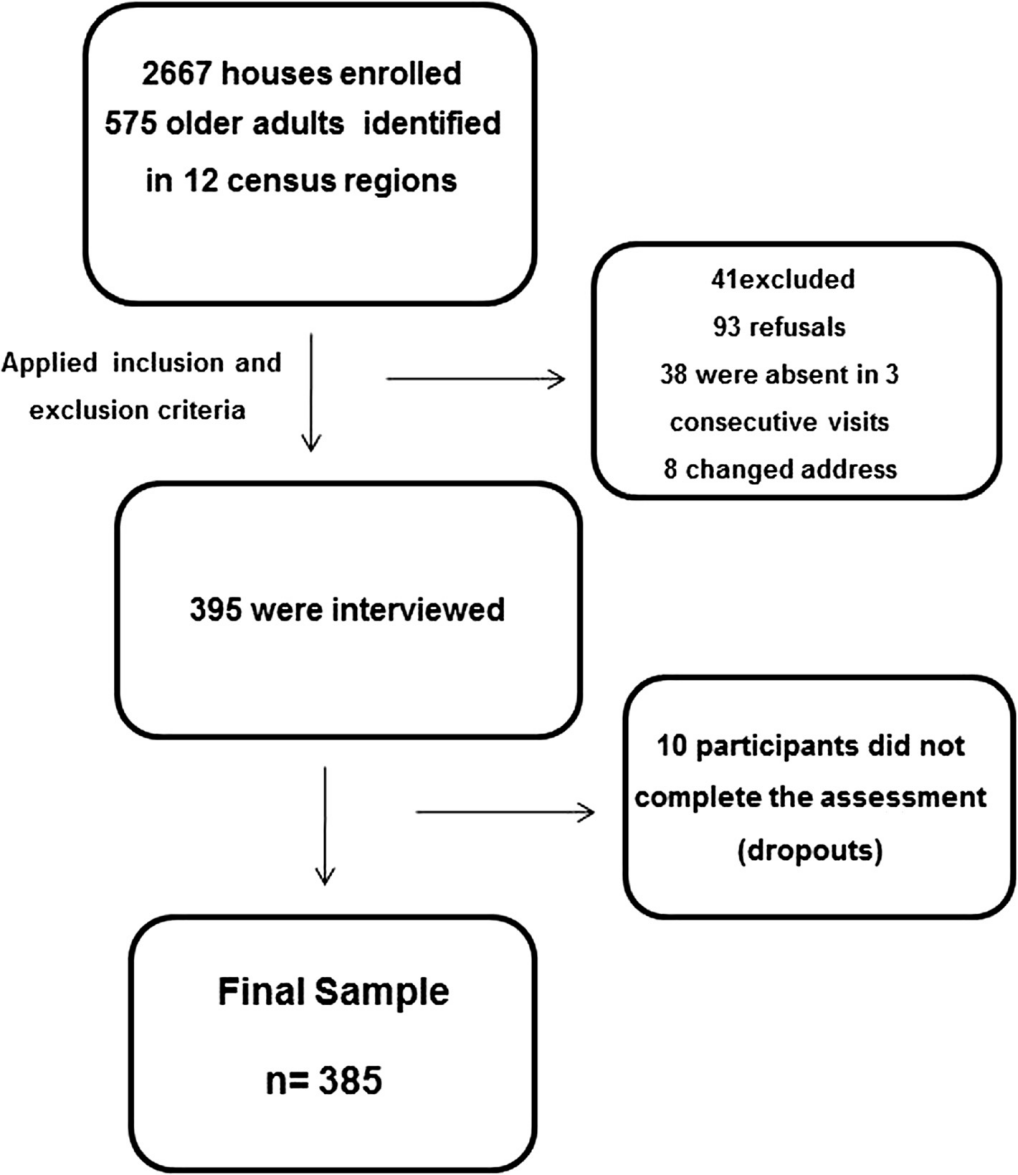
Flowchart of study sample.

The mean (SD) age of the 385 participants included in the study was 71.4 (5.7) years, ranging from 65 to 92 years and 64.4% were female. The vast majority of the participants had four or less years of education (82%) and the illiterates composed 30%. In terms of ethnicity, 47.8% of the participants considered themselves white, 37.1% as pardo or brown, 11.2% as black, and 3.9% as yellow or indigenous. A flow chart of the study sample is presented in Figure 1.

The mean (SD) usual gait speed was 1.11 (0.27) m/s and the mean fast gait speed was 1.39 (0.34) m/s. The percentage of participants with a usual gait speed below 1.0 m/s was 28.1%. The general characteristics, and the characteristics of the slower, intermediate and faster participants in terms of gait speed at usual and fast pace, are presented in Table 1.

**Table 1:** Usual gait speed and fast gait speed groups, sub-divided into slower, intermediate and faster groups (n = 385)

			**Usual gait speed**				**Fast gait speed**		
**Characteristics**	**General**	**Slower**	**Intermediate**	**Faster**	***p***	**Slower**	**Intermediate**	**Faster**	***p***
	**(N=385)**	**(N=75)**	**(N=207)**	**(N=103)**		**(N=58)**	**(N=218)**	**(N=109)**	
**Demographics**									
Age, years	71.4 (5.7)	75.0 (7.1)	70.8 (5.0)	69.8 (4.7)	<0.001	74.7 (6.6)	71.2 (5.4)	69.9 (4.8)	<0.001
Female, n (%)	248 (64.4)	52 (69.3)	151 (72.9)	45 (43.7)	<0.001	45 (77.6)	158 (72.5)	45 (41.3)	<0.001
Illiterate, n (%)	111 (28.9)	29 (38.7)	61 (31.8)	21 (20.4)	0.028	22 (37.9)	69 (31.8)	20 (18.3)	0.011
Income level									
0.0–1.0	189 (51.5)	41 (56.9)	109 (55.3)	39 (39.8)	0.103	32 (59.3)	115 (55.6)	42 (39.6)	0.003
1.1–3.0	128 (34.9)	23 (31.9)	64 (32.5)	41 (41.8)		19 (35.2)	70 (33.8)	39 (36.8)	
≥ 3.1	50 (13.6)	8 (11.1)	24 (12.2)	18 (18.4)		3 (5.6)	22 (10.6)	25 (23.6)	
Race/ethnicity, n (%)									
White	184 (47.8)	37 (49.3)	105 (50.7)	42 (40.8)	0.531	28 (48.3)	106 (48.6)	50 (45.9)	0.499
Black	43 (11.2)	11 (14.7)	18 (8.7)	14 (13.6)		10 (17.2)	21 (9.6)	12 (11.0)	
Pardo or brown	143 (37.1)	24 (32.0)	76 (36.7)	43 (41.7)		17 (29.3)	85 (39.0)	41 (37.6)	
**Mental health**									
MMSE score, points	23.9 (3.5)	22.2 (3.7)	23.9 (3.3)	25.1 (3.3)	<0.001	22.1 (3.7)	23.7 (3.3)	25.2 (3.1)	<0.001
Depressive symptoms, n (%)	76 (19.7)	17 (22.7)	47 (22.7)	12 (11.7)	0.001	17 (29.3)	43 (19.7)	16 (14.7)	0.077
High concern of falling, n (%)	192 (49.9)	55 (73.3)	109 (52.7)	28 (27.2)	<0.001	43 (74.1)	118 (54.1)	31 (28.4)	<0.001
**Physical health**									
Heart disease, n (%)	77 (20)	19 (25.3)	42 (20.3)	16 (15.5)	0.269	40 (69.0)	49 (22.5)	16 (14.7)	0.249
Hypertension, n (%)	249 (64.7)	52 (69.3)	135 (65.2)	62 (60.2)	0.44	8 (13.8)	150 (68.8)	59 (54.1)	0.025
Stroke, n (%)	28 (7.3)	11 (14.7)	13 (6.3)	4 (3.9)	0.017	22 (37.9)	17 (7.8)	3 (2.8)	0.029
Diabetes, n (%)	110 (28.6)	33 (44.0)	56 (27.1)	21 (20.4)	0.002	21 (36.2)	66 (30.3)	22 (20.2)	0.038
Arthritis, n (%)	89 (23.1)	27 (36.0)	48 (23.2)	14 (13.6)	0.002	4 (6.9)	55 (25.2)	13 (11.9)	0.001
Lung disease, n (%)	37 (9.6)	9 (12.0)	18 (8.7)	10 (9.7)	0.714	23 (39.7)	23 (10.6)	10 (9.2)	0.685
Urinary incontinence, n (%)	72 (18.8)	29 (38.7)	34 (16.4)	9 (8.8)	<0.001	33 (56.9)	37 (17)	12 (11)	<0.001
“Very good” and “Good” self rated health, n (%)	203 (52.7)	33 (44.0)	107 (51.7)	63 (61.2)	0.15	24 (41.4)	116 (53.2)	63 (57.8)	0.086
Two or more falls, n (%)	59 (15.3)	17 (22.7)	35 (16.9)	7 (6.8)	0.01	30 (51.7)	34 (15.6)	9 (8.3)	0.004
Single fall, n (%)	153 (39.7)	33 (44.0)	85 (41.1)	35 (34)	0.342	2.95 (1.51)	86 (39.4)	37 (33.9)	0.081
Three or more diseases, n (%)	140 (36.6)	43 (58.1)	75 (36.4)	22 (21.6)	<0.001	33 (56.9)	84 (38.9)	23 (21.3)	<0.001
Polypharmacy	133 (34.5)	39 (52.0)	70 (33.8)	24 (23.3)	<0.001	30 (51.7)	80 (36.7)	23 (21.1)	<0.001
Fatigue, n (%)	43 (11.2)	12 (16.0)	27 (13.0)	4 (3.9)	0.018	10 (17.2)	28 (12.8)	5 (4.6)	0.023
**Physical functioning**									
	77 (20)	31 (41.3)	37 (17.9)	9 (8.7)	<0.001	25 (43.1)	38 (17.4)	14 (12.8)	<0.001
Low PAL									
Impaired ADL, n (%)	25 (6.6)	11 (15.1)	11 (5.3)	3 (3.0)	0.004	10 (17.9)	12 (5.5)	3 92.8)	0.001

Abbreviations: MMSE: Mini-Mental Score Examination; PAL: Physical Activity Level; ADL: Activities of Daily Living.

Income level is presented as monthly income measured in multiples of the minimum wage (US$ 290 in 2009).

Continuous and discrete variables were compared using one-way ANOVA.

Categorical variables were compared using the Chi-squared test.

*p*<0.05.

Table 2 shows the crude odds ratio for having a slower usual gait speed and a slower fast gait speed. The association between gender and fast gait speed did not remain significant when stature was included in the model. Stature was independently associated with both usual and fast gait speed and was used to adjust both models. The multivariate adjusted model is shown in Table 3. Older adults aged 75 and over (OR = 3.81; 95% CI: 1.89–7.67), with a low level of physical activity (OR = 2.24; 95% CI: 1.18–4.25), stroke (OR = 3.41; 95% CI: 1.31–8.86), diabetes (OR = 2.25; 95% CI: 1.20–4.19), urinary incontinence (OR = 1.98; 95% CI: 1.02–3.84), and a high concern of falling (OR = 2.27; 95% CI: 1.21–4.24) showed higher odds of presenting with a slower usual gait speed compared with those who had none of these characteristics (Hosmer and Lemeshow test = 0.114; AUC = 0.807, 95% CI 0.749–0.866; p< 0.001). The final model for fast gait speed showed that an age of 75 or over (OR = 2.69; 95% CI: 1.29–5.62), low physical activity level (OR = 2.24; 95% CI: 1.15–4.36), urinary incontinence (OR = 2.44; 95% CI: 1.23–4.84) and a high concern of falling (OR = 2.30; 95% CI: 1.17–4.54) were associated with a slower fast gait speed (Hosmer and Lemeshow test = 0.403; AUC = 0.789, 95% CI 0.721–0.858; p< 0.001).

**Table 2:** Crude odds ratio for slower usual and slower fast gait speeds (n=385)

**Variables**	**Usual gait speed**		**Fast gait speed**	
	**OR (95% IC)**	***p***	**OR (95% IC)**	***p***
**Demographics**				
Female	1.29 (0.75-2.22)	0.359	2.12 (1.10-4.09)	0.024‡
Age group (years)				
70-74 (ref 65–69)	1.07 (0.53-2.18)	0.846	1.58 (0.74-3.36)	0.233
75 and over	3.91 (2.16-7.06)	<0.001‡	3.63 (1.86-7.07)	<0.001‡
Illiterates (ref >1 year of schooling)	1.74 (1.02-2.96)	0.039‡	1.62 (0.90-2.91)	0.102
Income level				
1.1-3.0 (ref 0.0-1.0)	0.79 (0.44-1.39)	0.418	0.85 (0.46-1.58)	0.620
3.1 or over (ref 0.0-1.0)	0.68 (0.29 – 1.57)	0.377	0.31 (0.09-1.06)	0.064
**Mental health**				
MMSE score	0.85 (0.79-0.91)	<0.001‡	0.84 (0.77-0.91)	<0.001‡
	1.27 (0.69-2.34)	0.445	1.87 (1.00-3.53)	0.051
Depressive symptoms				
High concern of falling	3.65 (2.07-6.45)	<0.001‡	3.40 (1.82-10.53)	<0.001‡
**Physical health**				
Hypertension	1.27 (0.74-2.19)	0.386	1.26 (0.69-2.30)	0.450
Heart diseases	1.40 (0.76-2.55)	0.278	1.07 (0.53-2.13)	0.852
Stroke	3.01 (1.34-6.73)	0.007‡	2.44 (1.02-5.86)	0.044‡
Diabetes	2.38 (1.41-4.01)	0.001‡	1.65 (0.92-2.96)	0.092
Arthritis	2.25 (1.30-3.89)	0.004‡	2.15 (1.18-3.91)	0.012‡
Lung disease	1.37 (0.61-3.04)	0.441	0.65 (0.22-1.92)	0.442
Urinary incontinence	3.98 (2.26-7.03)	<0.001‡	3.70 (2.01-6.79)	<0.001‡
Number of diseases				
1 or 2 diseases (ref 0 disease)	1.83 (0.60-5.50)	0.282	3.17 (0.72-13.97)	0.126
≥ 3 diseases (ref 0 disease)	4.98 (1.68-14.74)	0.004‡	7.24 (1.67-31.45)	0.008‡
Polypharmacy (ref 0–3 drugs)	2.42 (1.45-4.06)	<0.001‡	2.35 (1.33-4.14)	0.003‡
Fatigue	1.74 (0.85-3.58)	0.131	1.85 (0.85-4.00)	0.118
Poor self-rated health (ref good or very good)	1.54 (0.93-2.56)	0.093	1.71 (0.97-3.01)	0.062
Single fall (ref no falls)	1.27 (0.76-2.13)	0.353	1.77 (1.01-3.10)	0.047‡
Two or more falls (ref no falls)	1.90 (1.01-3.58)	0.046‡	2.51 (1.30-4.85)	0.006‡
**Physical functioning**				
≥ 1ADL ( ref 0 ADL)	3.77 (1.63-8.71)	0.002‡	4.46 (1.89-10.53)	0.001‡
Low physical activity level (ref other quintiles)	3.91 (2.23-6.85)	<0.001‡	4.08 (2.24-7.44)	<0.001‡

Abbreviations: ref, reference category; OR, Odds ratio; CI, Confidence Interval; MMSE, Mini-Mental State Examination; ADL, Activities of Daily Living.

ORs denote the odds of having a slower usual gait speed or a slower fast gait speed.

‡p-values that included variables used in the multivariate analysis (*p*<0.05).x.

**Table 3:** Multivariate adjusted model for slower usual and slower fast gait speeds

**Variables**	**Usual gait speed**		**Fast gait speed**	
	**OR (95% IC)**	***p***	**OR (95% IC)**	***p***
Age group (years)				
70-74 (ref 65–69)	1.01 (0.47-2.18)	0.966	1.51 (0.68-3.38)	0.308
≥ 75 (ref 65–69)	3.81 (1.89-7.67)	<0.001	2.69 (1.29-5.62)	0.008
Low PAL	2.24 (1.18-4.25)	0.013	2.24 (1.15-4.36)	0.017
Stroke	3.41 (1.31-8.86)	0.012		
Diabetes	2.25 (1.20-4.19)	0.011		
Urinary incontinence	1.98 (1.02-3.84)	0.041	2.44 (1.23-4.84)	0.010
High concern of falling (FESI)	2.27 (1.21-4.24)	0.010	2.30 (1.17-4.54)	0.016

Abbreviations: ref, reference category; OR, Odds ratio; CI, Confidence Interval; PAL, Physical Activity level; FESI, Falls Efficacy.

Scale International.

ORs represent the odds of having a slower usual gait speed and a slower fast gait speed.

The model was adjusted for height.

## Discussion

This study is among the first to investigate gait speed (both usual and fast) in a large and representative sample of community-dwelling older adults living in a developing country. Neither usual gait speed nor fast gait speed showed an independent association with any social demographic outcomes (other than age) such as race/ethnicity, income, or educational level, suggesting that health status might be the main contributor to a slower gait speed in this population.

The odds of walking at a slower usual or fast usual speed increase with age. Regarding specific health conditions, urinary incontinence, stroke, and diabetes were the main contributors to a slower usual gait speed, and urinary incontinence largely influenced fast usual gait speed. Older adults with a low perceived self-efficacy manifested by a high concern of falling and a low level of physical activity had higher odds of being in the slower usual gait speed or slower fast gait speed groups.

### Comparison with previous studies

The average gait speed at usual pace, mean age, and race/ethnicity characteristics observed in our study were similar to those observed in US studies [2,34], in which the population showed a mean usual gait speed of 1.12 m/s. The participants’ mean age was 73.6 years, and the population comprised whites (58.5%) and blacks (41.5%). Also, the percentage of individuals with a usual gait speed below 1.0 m/s was quite similar [2,34]. Two studies [2,35], which included only men, and another with a European population [16], showed similar mean gait speed values to those presented in the present study; however, the vast majority of the sample comprised white participants. Interestingly, comparing our results with those obtained in another US study involving only a Hispanic population shows that the difference in mean usual gait speed was substantial (1.11 m/s *vs.* 0.56 m/s, respectively), and the prevalence of a usual gait speed below 1.0 m/s in this Hispanic population was at least three times higher (95.6%) [2] than that reported in the present study. We did not observe racial differences in gait speed. However, a recent study [21] that compared Caucasians and African Americans identified that gait speed was slower in African Americans, even when adjusting for multiple confounders and covariates, such as age, gender, education, comorbidities and pain. One possible explanation is that, in some multiracial samples, such as ours, the ambiguity in racial classification can be substantial [36], mainly in Brazil where race is based primarily on skin colour rather than ancestry. We overcome part of this problem using a self-classification method instead of an interviewer classification method that has been proven to be less biased by socioeconomic position [36], however, other factors in our study may have possibly influenced the lack of association between gait speed and race, such as personal and environmental factors.

In previous studies, socioeconomic inequalities such as high financial insecurity levels, low employment and low educational levels were reported as important contributors to reduced gait speed in older adults [23,24]. These associations were partly explained by differences in health behaviors and incidence of chronic diseases largely explained by physiological measures that have an impact in physical function, particularly related to lower extremity disability among older adults. In addition, our study did not confirm an independent association between socioeconomic status outcomes and gait speed. Lower educational level was crudely associated with slower usual gait speed but this association became non-significant when adjusted by age, mobility-related disorders and fear of falling. Moreover there was a high number of participants in low educational and low-income strata in our sample, which might have attenuated the influence of social disparities in gait speed.

We identified a strong independent association between age and usual and fast gait speeds. The mean age was higher in the slower groups. This finding is corroborated by other studies [1,2] and can be explained by the adoption of a more conservative basic gait pattern, which is likely to be a compensatory strategy to maintain balance in the presence of age-related deficits in physiological function [37].

Slow usual gait speed was associated with neuromuscular mobility-related disorders. Diabetes is considered to be a subclinical inflammatory condition that contributes to the aetiology of metabolic and cardiovascular complications, and is associated with sarcopenia [38], which is an early indicator of functional decline [39]. The literature shows that patients with diabetes walk more slowly and have greater variability in stride length [40]. In addition, older patients with diabetes show abnormal functional balance and mobility-related disabilities, which in turn can compromise gait speed [41]. Stroke is commonly associated with slower cadence, shorter stride length and weakness of hip flexors and knee extensors, which ultimately reduce gait speed [42,43].

Global cognitive function was crudely associated with slower usual and fast gait speeds, but was not associated in the final regression models. However, studies show a relationship, both cross-sectionally and longitudinally, between gait speed and cognitive function [8,9]. The lack of association identified in the present study may be explained, in part, by the fact that we excluded those with a severe cognitive decline.

Older adults that are less confident in their balance control tend to change the temporospatial parameters of gait, such as adopting a reduced stride length, and an increased stance width and double support time [44,45]. It is suggested that older people who are afraid of falling, or have a lower perceived self-efficacy, develop a more hesitant motor control pattern, shifting their control of balance from an automatic fast mode to a more conscious, slow mode, thereby compromising their anticipatory postural adjustments, which might explain why they select a slower gait speed [44]. It is also noteworthy that we found a crude association between gait speed and recurrent falls, highlighting the vicious cycle of falls, fear of falling, and poor physical functioning.

Physical activity level and urinary incontinence were independently associated with a slow usual gait speed and a fast usual gait speed, which suggests a rationale that goes beyond the cumulative effect of certain disease burdens. Older people seem to self-select walking speed according to their functional reserves, and some studies show that more sedentary behaviour compromises maximal oxygen uptake [10,46] which, in turn, contributes to slow walking speed [1,47]. Regarding the relationship between urinary incontinence and slower walking speed, we suggest that they might share common physiological pathways, since both activities must rely on good muscular function, which is not only related to strength but also to a proper automatic muscle response that works on a “demand” basis [48]. Other than that, urinary incontinence can be influenced by the perceived self-efficacy for avoiding urine leakage while walking [49].

### Study limitations

This study has limitations imposed by its cross-sectional design, which did not allow us to establish causal links. In addition, the presence of diseases was assessed by self-reporting, which may result in over or under-estimation of disease prevalence. However, we asked participants to report only those conditions diagnosed by a physician; hence, we do not expect that this affected the results substantially. We excluded older adults with severe cognitive decline, and also those with severe neurological conditions, which might limit the external validity of the study. However, we ensured that we covered all the selected census areas and tried to guarantee that all older adults living in the area were interviewed. Additional streets in the same region were selected to compensate for drop-outs and to maintain the cluster sampling.

Considering gait speed as a strong marker of overall health status and mortality in older adults [15], our current results shows that some interventions that may prevent a decrease in gait speed can be carried out in large populations. These include improvements in physical activity levels. On an individual basis, the assessment of gait speed in specific groups of older people (such as those with neuromuscular mobility-related disorders) may provide useful information that can be used for further comprehensive geriatric assessments. The prevalence of incontinence is increasing in both women and men [50] and its management in the elderly is frequently neglected, despite its well-known association with poor quality of life and psychological wellbeing. Evidence is accumulating that all conservative management strategies used in younger adults can be used in selected older, motivated people, including life style modifications, pelvic floor muscle training for those with stress incontinence, and bladder retraining for those with urge incontinence [51]. In addition, interventions aimed at reducing activity avoidance by older adults with a high fear of falling may help to prevent associated decreases in gait speed.

## Conclusion

The results of the present study show that a multiracial population of older adults living in an urban area in a developing country tends to self-select its gait speed in a manner similar to older adults in US and European populations. Older participants and those with mobility-related disorders seem to self-select a slower usual gait speed. A lower level of perceived self-efficacy, manifested as a high fear of falling, urinary incontinence, and low physical activity levels, seems to be an important constraint on both usual and fast gait speed, and its causal links should be further investigated. Considering gait speed as an overall marker of health status, our results highlight the fact that these conditions should not be neglected.

## Competing interests

The authors declare no conflicts of interest.

## Authors’ contributions

CRR, TLB, LFT, JLAR, SRA, RCD, MRP were responsible for data acquisition and data analysis and interpretation, drafting the article and final approval of the manuscript. MRP and TLB were local coordinators of the multicentre study and also responsible for general supervision of the research group. RCD was one of the coordinators for the FIBRA Study nationally and responsible for the acquisition of funding. All authors read and approved the final manuscript.

## Authors’ information

TLB, RCD, MRP are chief investigators in a Research Network, named Rede FIBRA, funded by the Brazilian government, which studies the determinants of frailty and other health-related conditions in older adults living in cities with different development indexes.

## Pre-publication history

The pre-publication history for this paper can be accessed here:

http://www.biomedcentral.com/1471-2458/13/182/prepub
